# Cronkhite-Canada Syndrome With Advanced Gastric Cancer

**DOI:** 10.14309/crj.0000000000001328

**Published:** 2024-06-19

**Authors:** Atsushi Goto, Shinichi Hashimoto, Kazuhiro Yamamoto, Jun Nishikawa, Taro Takami

**Affiliations:** 1 Department of Gastroenterology and Hepatology, Yamaguchi University Graduate School of Medicine, Ube, Japan; 2 Faculty of Laboratory Science, Yamaguchi University Graduate School of Medicine, Ube, Japan

## CASE REPORT

A 60-year-old man was diagnosed 11 years ago as having Cronkhite-Canada syndrome (CCS) based on multiple gastric polyps, diarrhea, hypoalbuminemia, and skin symptoms. There were multiple hamartomatous polyps in the colon but no polyps in the small intestine. After starting oral prednisolone (30 mg/day) 6 years ago, some polyps had reduced in size, but a protruding lesion of the gastric antrum tended to increase.

**Figure 1.**
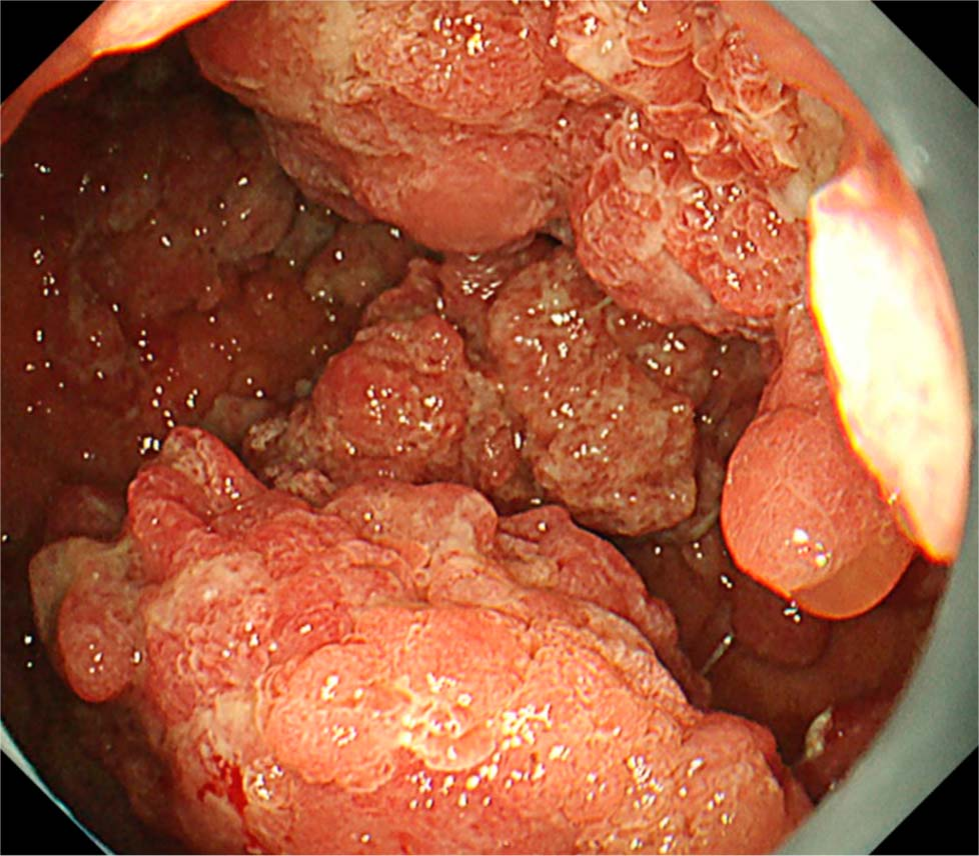
Esophagogastroduodenoscopy showed a mixture of typical red caviar-like non-neoplastic polyps and a lesion of suspected neoplasticity.

**Figure 2.**
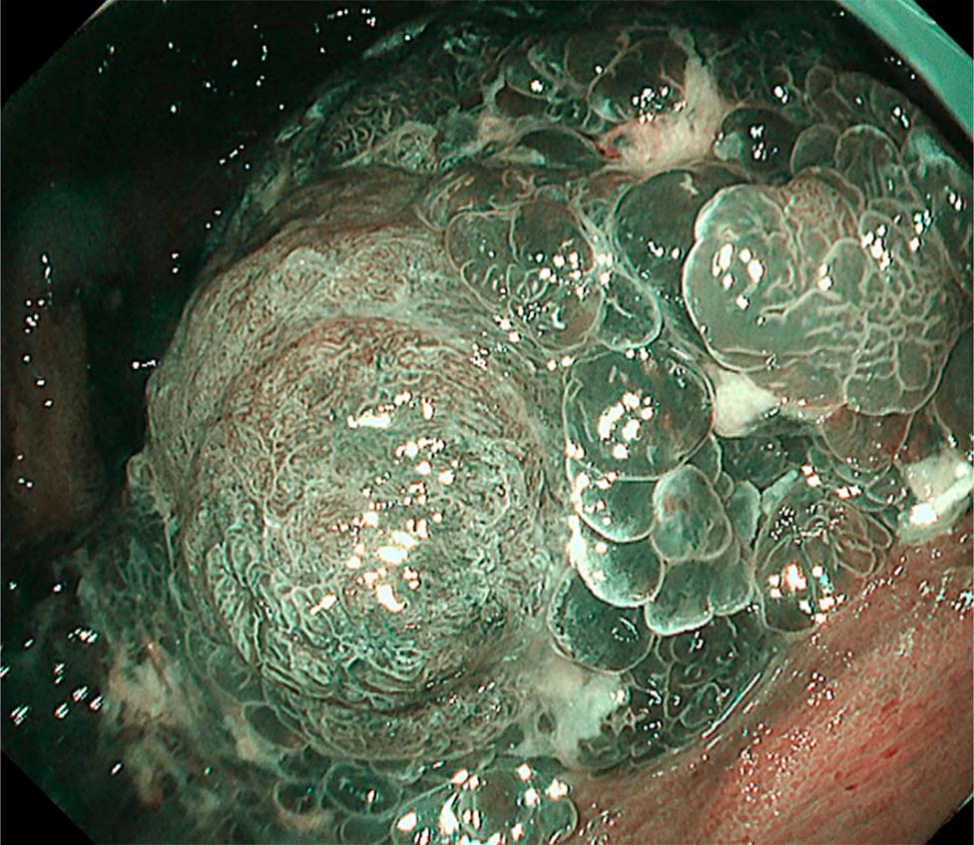
Narrow-band imaging clarified irregular surface-patterned lesion suggestive of neoplasticity.

Routine esophagogastroduodenoscopy showed a mixture of typical red caviar-like non-neoplastic polyps with edema and hyperemia and a protruding lesion with irregular surface pattern suspected of being a neoplasticity (Figures 1 and 2). Biopsy revealed irregular tubular structures suspicious for gastric cancer, so laparoscope-assisted distal gastrectomy was performed.

**Figure 3.**
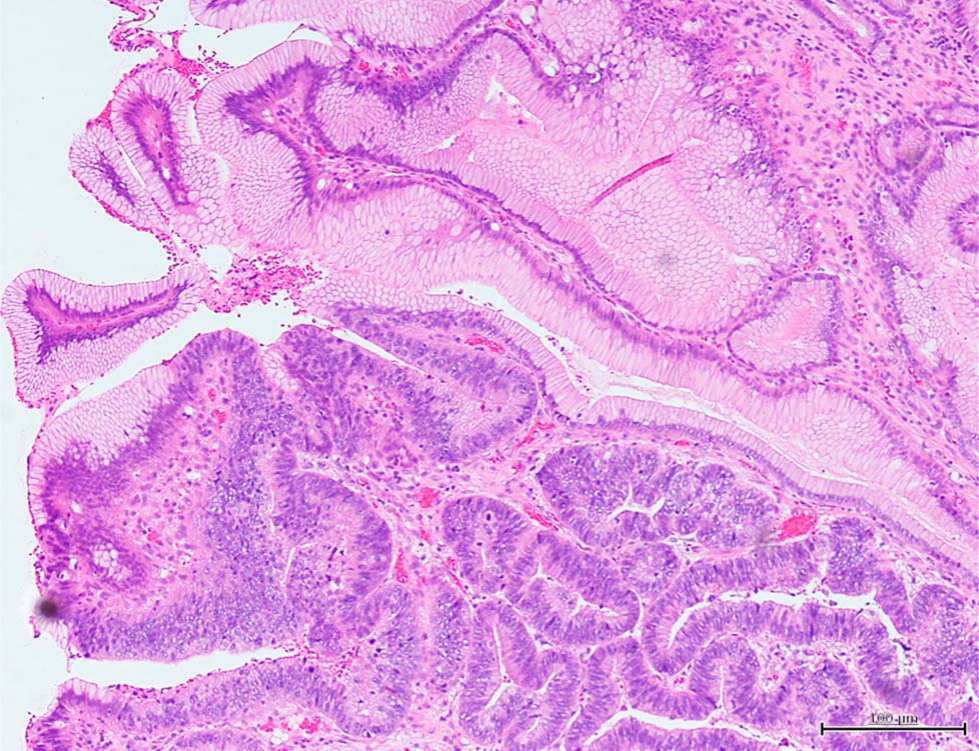
Surgical specimen with mixed non-neoplastic tissue and tubular adenocarcinoma (100× magnification).

**Figure 4.**
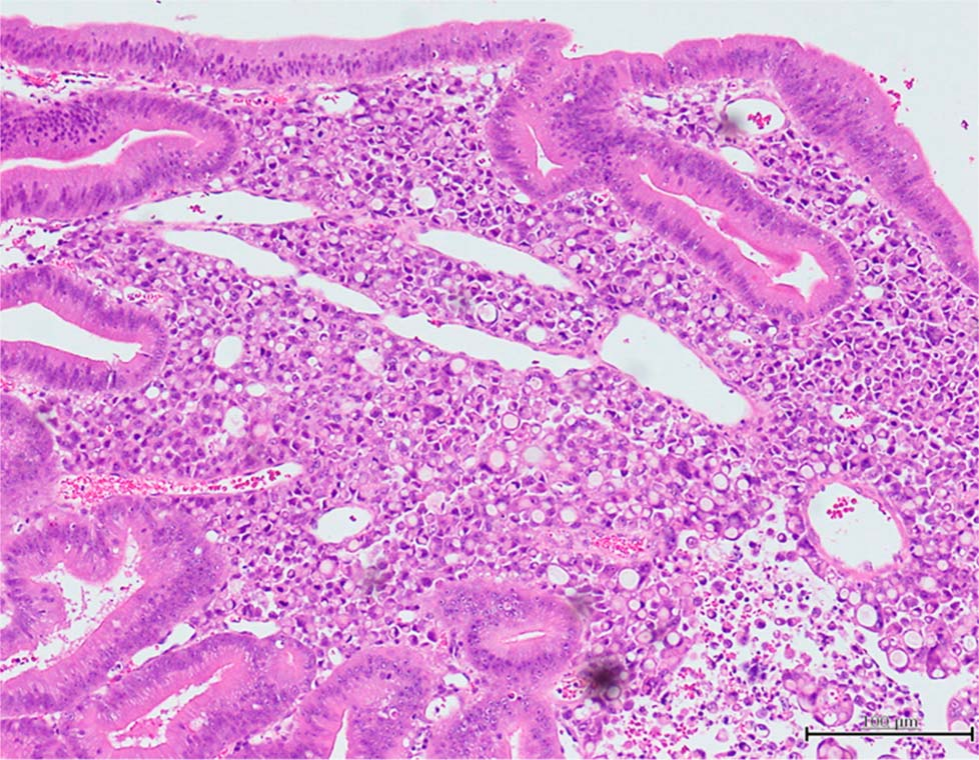
Surgical specimen with mixed signet-ring cell carcinoma and adenoma (100× magnification).

Surgical specimens showed a mixture of non-neoplastic tissue characterized by stromal edema and dilated glandular ducts and diverse neoplastic tissue such as tubular adenocarcinoma, signet-ring cell carcinoma, and adenoma (Figures 3 and 4). Chemotherapy was begun because the cancer had invaded the subserosa and metastasized to numerous lymph nodes. The patient died 1 year and 2 months after starting chemotherapy.

CCS is a rare gastrointestinal polyposis syndrome diagnosed on the basis of gastrointestinal symptoms such as chronic diarrhea and skin symptoms such as alopecia, onychodystrophy, and pigmentation. CCS requires caution because of complications of adenoma and carcinoma. In Japan, the gastric cancer complication rate for CCS is reported to be 10.2%.^1^ Durable polyp regression is associated with decreased cancer risk. Oral prednisolone at doses of 30 mg/day or more is appropriate for inducing clinical remission and regression of polyps.^2^ Endoscopic screening is also recommended at least once a year.^1^

The *Helicobacter pylori* antibody in this case was negative. The surgical specimens showed a mixture of non-neoplastic and neoplastic tissue, which strongly suggests that the cancer originated from a CCS polyp. Although this patient had advanced cancer, we believe this to be a valuable case in which preoperative observation with narrow-band imaging revealed the complication of neoplastic lesions.

## DISCLOSURES

Author contributions: A. Goto: writing-original draft. S. Hashimoto: creating images. K. Yamamoto: creating images. J. Nishikawa: writing-original draft. T. Takami: supervision. A. Goto is the article guarantor.

Financial disclosure: None to report.

Previous presentation: Yamaguchi University Gastrointestinal Conference; November 29, 2023; Yamaguchi University Hospital.

Informed consent was obtained for this case report.
